# Electronic and transport properties of kinked graphene

**DOI:** 10.3762/bjnano.4.12

**Published:** 2013-02-15

**Authors:** Jesper Toft Rasmussen, Tue Gunst, Peter Bøggild, Antti-Pekka Jauho, Mads Brandbyge

**Affiliations:** 1 Center for Nanostructured Graphene (CNG), Department of Micro- and Nanotechnology (DTU Nanotech), Technical University of Denmark, DK-2800 Kongens Lyngby, Denmark

**Keywords:** adsorption and reactivity, curvature effects, DFT calculations, electronic transport, graphene nanoribbons, graphene nanostructuring

## Abstract

Local curvature, or bending, of a graphene sheet is known to increase the chemical reactivity presenting an opportunity for templated chemical functionalisation. Using first-principles calculations based on density functional theory (DFT), we investigate the reaction barrier reduction for the adsorption of atomic hydrogen at linear bends in graphene. We find a significant barrier lowering (≈15%) for realistic radii of curvature (≈20 Å) and that adsorption along the linear bend leads to a stable linear kink. We compute the electronic transport properties of individual and multiple kink lines, and demonstrate how these act as efficient barriers for electron transport. In particular, two parallel kink lines form a graphene pseudo-nanoribbon structure with a semimetallic/semiconducting electronic structure closely related to the corresponding isolated ribbons; the ribbon band gap translates into a transport gap for electronic transport across the kink lines. We finally consider pseudo-ribbon-based heterostructures and propose that such structures present a novel approach for band gap engineering in nanostructured graphene.

## Introduction

Nanostructures based on graphene have an enormous potential for applications. Especially in future electronic devices compatible with and extending silicon technology, due to the outstanding electronic transport properties of graphene [1]. However, it is crucial to modify the semimetallic electronic structure of graphene to exploit its full potential for many electronic applications: a band gap can be introduced by nanostructuring graphene.

A common approach towards engineering the electronic structure is to form quasi-1D graphene in the form of nanoribbons (GNR) [2]. The electronic structure of GNRs depends on width, direction and edge structure – all parameters that to some degree can be controlled. GNRs can be formed by etching [2], by unzipping carbon nanotubes (CNTs) [3], or ultimately be grown with atomic-scale precision by using self-assembly of precursor molecules on metal substrates [4]. However, for electronic applications this approach requires a structure-preserving means of releasing and transferring the structures to an insulating substrate. Bonding of H or other species to graphene with large coverage opens an insulating band gap at the adsorption sites due to sp^3^ hybridisation [5]. Periodically ordered clusters of adsorbed hydrogen can be formed on graphene in patterns dictated by the Moiré lattice mismatch between graphene and the metal substrate, which opens a semiconducting band gap [6]. Finally, regular perforations, known as a graphene antidot lattice (GAL) [7], or a nanoscale mesh of holes [8–10] can have neck widths [10–11] down to 5 nm corresponding to band gaps of the order of 1 eV [2].

Graphene consists entirely of surface atoms and is thus exceedingly sensitive to the surroundings. In particular, the van der Waals (vdW) interaction with the substrate is of importance. The substrate interactions, which make graphene cling to small features, may be exploited by manufacturing nanostructures in the substrate. Periodic steps in a Cu substrate has been used to induce “wrinkles” or ripples in graphene with period and height on the order of 10 nm [12]. Recently, Hicks et al. [13] demonstrated how arrays of 1D large band gap, semiconducting graphene nanoribbons corresponding to a width of ≈1.4 nm can be formed in graphene on a step-patterned SiC substrate. The substrate interactions can clamp a graphene sheet while partly suspended across small holes, so that a pressure difference between the in- and outside leads to the formation of bubbles or “blisters” in the sheet [14]. Also, linear folds, where the graphene sheet is bulging up from the substrate, have been induced for graphene suspended over trenches by using heat treatment [15]. Thus, the sheet can obtain significant bends at certain places induced by the substrate interaction, substrate nanostructuring, and subsequent treatments [16]. Calculations by Low et al. [17] showed how a sharp step of height 1 nm in a SiC substrate, comparable to experimental values [13], can induce a linear bend in the graphene sheet with a radius of curvature down to around 1 nm.

The ability to accurately control sharp local curvatures of graphene presents opportunities for strain-assisted modification of the local electronic structure and chemical reactivity in the graphene sheet, and may open a route to band gap engineered devices [13,18–21]. Very recently, Wu et al. [21] showed how graphene on a Si substrate decorated with SiO_2_ nanoparticles induced local regions of strain and increased reactivity in a selective manner. Atomic hydrogen or other chemical species do not easily react with flat graphene when dosed from a single side [22]. However, at positions where there is a substantial local bending, rippling or strain of the graphene sheet the reactivity changes significantly [5,23]. So far there have been only a few theoretical studies of the atomic geometry of hydrogenated ripple structures in unsupported, strain-induced, graphene ripples [24–26]. However, to the best of our knowledge, no studies have addressed the reactivity of bends or the transport through hydrogenated ripples, or discussed the possibility of stabilising nonplanar structures by hydrogenation.

**Figure 1:**
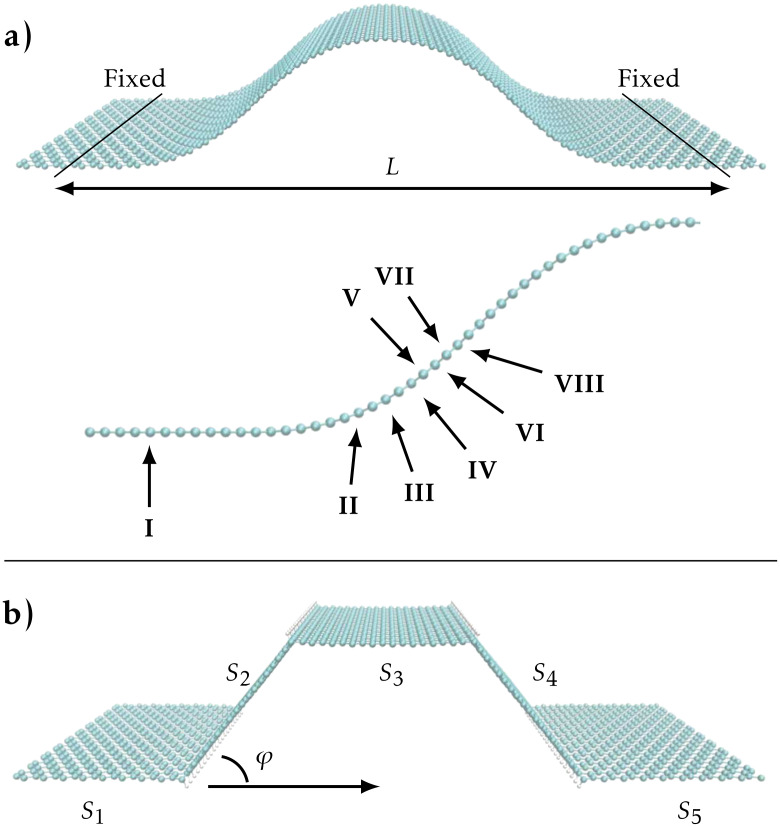
(a) Smooth ripple-like structure where the first and last six rows of carbon-dimers are surface-clamped regions with a separation of *L* = 90 Å. Atomic hydrogen is adsorbed at positions I–VIII. (b) The resulting kinked graphene structure after hydrogen is adsorbed in lines at the most reactive position (II) corresponding to the smallest local radius of curvature. The four kinks divide the structure into five sections, *S*_1_–*S*_5_.

In this paper we consider the reactivity of linear bends in a graphene sheet, and the electronic transport properties of kinks resulting from the hydrogenation of bends. Our starting point is the generic graphene structure shown in Figure 1a, which is inspired by the experimental observation of trench formation [15]. The bulging of this structure results from shortening the distance between two separated, clamped regions in the sheet. The remaining sections of the paper are organised as follows: Section “System setup” describes our computational method and setup. In the subsequent section we present our results. First, we describe the adsorption barriers for the reaction with single atomic H on the graphene bend at positions with different local curvature (positions I–VIII in Figure 1a). Then we show how a linear bend transforms into a kink when decorated by H along the most reactive (most curved) line (Figure 1b), and we present the electronic transmission through a single kink in the subsection “Single kink”. The kink acts as an effective barrier with its transmission depending on the kink-angle, φ. In the subsection “Two kinks” we study how two parallel kinks lead to the formation of a pseudo-ribbon-type electronic structure. Finally, in subsection “Multiple kinks” we demonstrate the opening of a transport gap for multikink systems, such as the one shown in Figure 1b.

## System setup

The bend we consider in Figure 1a is created along the armchair direction by fixing the first and last six rows of carbon atoms and shortening the separation *L*, while the rest of the atoms are allowed to relax. A separation of *L* = 90 Å is chosen in order to obtain realistic curvatures [17]. We first assessed the reactivity of the structure at positions with different local curvature, see positions I–VIII in Figure 1a. Subsequently we relaxed the structure where lines of hydrogen (Figure 1b) have been placed at the points of lowest radius of curvature, i.e., the points of highest local reactivity. This particular system is meant to illustrate the potential of the hydrogen adsorption mechanism, and to gain insight into the modification of the electronic properties due to the hydrogen lines. In a corresponding experimental setup we can imagine placing graphene across a trench, which allows hydrogen adsorption on either side of the sheet.

The atomic and electronic structure calculations are based on density functional theory (DFT) using the SIESTA [27] code, and the PBE-GGA exchange-correlation [28] functional. We employ periodic boundary conditions (PBC) in the direction along the bend with a cell-width of four carbon dimers, and 10 Monkhorst–Pack *k*-points. We use a mesh cut-off of 500 Ry throughout. When calculating reactivity in the form of reduced reaction barriers the unit-cell is chosen so that the distance between single hydrogen atoms is larger than 8.5 Å. This ensures low hydrogen–hydrogen interaction, which is known to impact reaction barriers [29]. In the total energy calculations of relaxed atomic geometries and reaction barriers, we also use PBC transverse to the bend (5 *k*-points). We use a TZP basis-set for hydrogen and a SZ basis for carbon, except in the reaction barrier calculations where we compare calculations using a DZP and SZP basis for the four carbon atoms nearest to the hydrogen. In the barrier determinations we furthermore use spin-polarised calculations because of unpaired electrons. For the relaxed geometries a force tolerance of 0.01 eV/Å is used, and the final energies are corrected for basis-set-superposition errors (BSSE) [30].

Based on the computed atomic and electronic structures we subsequently use the TranSIESTA [31] method to calculate the electronic conductance per unit-cell width transverse to the bend. To this end we attach semi-infinite flat graphene electrodes to each side of the selected kinks, i.e., replace sections *S*_1_ and *S*_2_ in Figure 1b by semi-infinite electrodes in order to calculate the transmission through the single kink separating *S*_1_ and *S*_2_. In the conductance calculations we employ a dense transverse *k*-point grid of 400 points.

## Results and Discussion

### Adsorption barrier

Adsorption of hydrogen on graphene involves a reaction barrier that needs to be overcome before the single hydrogen atom sticks to the graphene sheet. Several investigations based on DFT calculations show that atomic hydrogen adsorbs on-top on flat graphene with a barrier about 0.2 eV and binding energy in the range of 0.7–1.0 eV [22,32–34]. Thus a minimum kinetic energy for the first hydrogen to react [32–33] is required, in an out-of-equilibrium situation such as in an atomic beam [34]. Casolo et al. [35] calculated the reaction barrier and adsorption energy for multiple hydrogen atoms on flat graphene. In agreement with other studies they found decreased barriers to sticking for the second H atom, compared to the barrier for adsorbing a single H atom on a clean surface [36].

Here, we first focus on the trends in the change in adsorption barrier as a function of the local curvature of the graphene sheet. To this end we have considered atomic hydrogen absorption at the on-top carbon positions at points with different curvature on the bent structure, see Figure 1a (positions I–VIII). The barrier is determined by calculating the total energy for each position of hydrogen above graphene as the hydrogen is moved successively closer to the graphene. Following the adsorption investigations on flat graphene by Ivanovskaya et al. [37] we perform, in each step, a relaxation of the hydrogen-bonded carbon atom and its three nearest neighbours. Using the method described above we obtain a reaction barrier of 0.22 eV on locally flat graphene. This is comparable to results obtained by several groups using DZP or plane wave basis sets and the PW91 functional [22,29,32–33]. We find that a SZP basis set for the relaxed carbon atoms yields a reduced barrier height of 0.18 eV (both basis sets with orbital range corresponding to an energy shift of 0.01 eV). Hence, we use the SZP basis in the following reaction-barrier calculations in order to lower the computation time.

**Figure 2:**
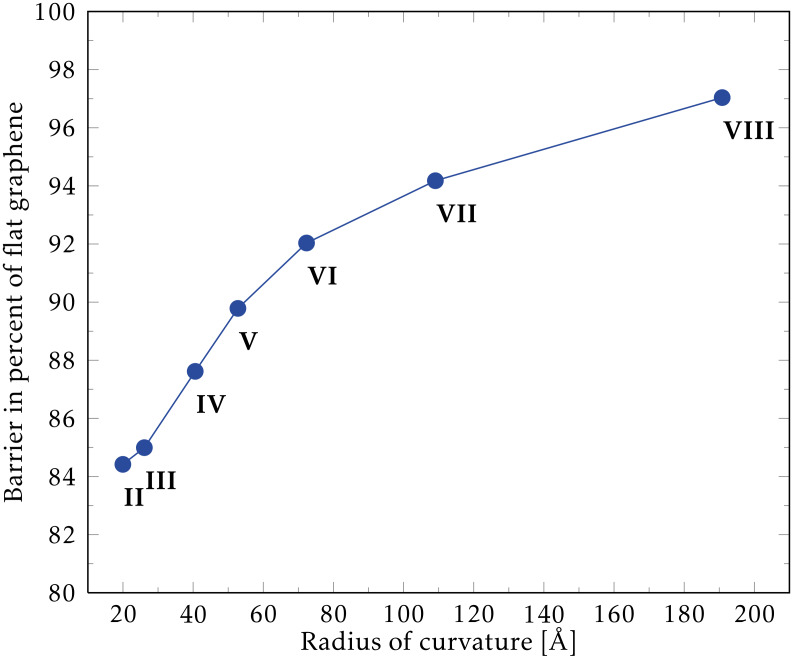
Calculated reaction barriers for hydrogenation of bent graphene as a function of the local radius of curvature (II–VIII in Figure 1a). Flat graphene (position I) has an infinite radius of curvature and is used to normalise the barriers. Calculations are spin-polarised and allow for atomic relaxation.

For the positions (I-VIII) we obtain the reaction barrier for adsorption of hydrogen as a function of the local radius of curvature (RoC) shown in Figure 2. The second least curved position (VIII), resulting in a large RoC, reduces the barrier by roughly 3% compared to flat graphene (position I). The most curved position (II) in our considered structure has a minimum RoC of ≈20 Å resulting in a barrier reduction of roughly 16%. For comparison, this RoC roughly corresponds to the radius of a (25,25) nanotube. Experiments by Ruffieux et al. [38] compare hydrogen adsorption on C_60_ molecules, CNTs, and graphite to show that reactivity is increased with curvature. In our case we find that the local electronic density of states changes little for the atoms on the pristine bent graphene sheet (as in Figure 1a). Thus, we conclude that the lowering of the adsorption barrier for the moderate RoC of about 20 Å is mainly due to the mechanical strain in the bend shifting the carbon atoms out of the graphene plane in the direction of the hydrogen. We note that an additional increase in reactivity may result from the change in electronic structure for highly bent graphene. Thus, we expect an increase in reactivity for the graphene with a linear bend, and a simple Arrhenius estimate using our data yields a factor of 3–4 at room temperature (300 K). We have also performed calculations using the less rigorous DFTB method [39] and obtained results in agreement with the trend in reaction-barrier reduction obtained above.

We may understand the reaction barrier and its change with curvature by considering the changes in carbon bond lengths. The barrier is due to the fact that the reacting carbon atom has to be pulled out of the graphene plane, stretching the strong carbon–carbon bonds, when reacting with the incoming hydrogen atom. When the graphene sheet is curved the carbon atom is already slightly out of the plane, and thus the energy required to pull the atom further out of plane is decreased compared to flat graphene. The ortho- and para-locations in the graphene hexagon have been shown to be the preferred locations for hydrogen adsorption in studies of flat graphene [22,29,40]. With this in mind as well as the curvature-related reduction of reaction barriers, we conclude that the considered system allows the adsorption of hydrogen atoms in single lines along armchair-edges. The kink in the atomic structure due to the sp^3^-binding of a single H makes the graphene curve even more in its vicinity, which in turn, preferentially lowers the barrier for absorption of a H along the linear bend. This suggests a mechanism in which the hydrogen adsorption is propagating and leads to the decoration of the entire linear bend turning it into a kink line. It may be viewed as analogous to crack-formation mechanisms, where the breaking of a bond increases the stress on neighbouring bonds; only in this case, the graphene is hydrogenated rather than broken or destroyed.

### Single kink

**Figure 3:**
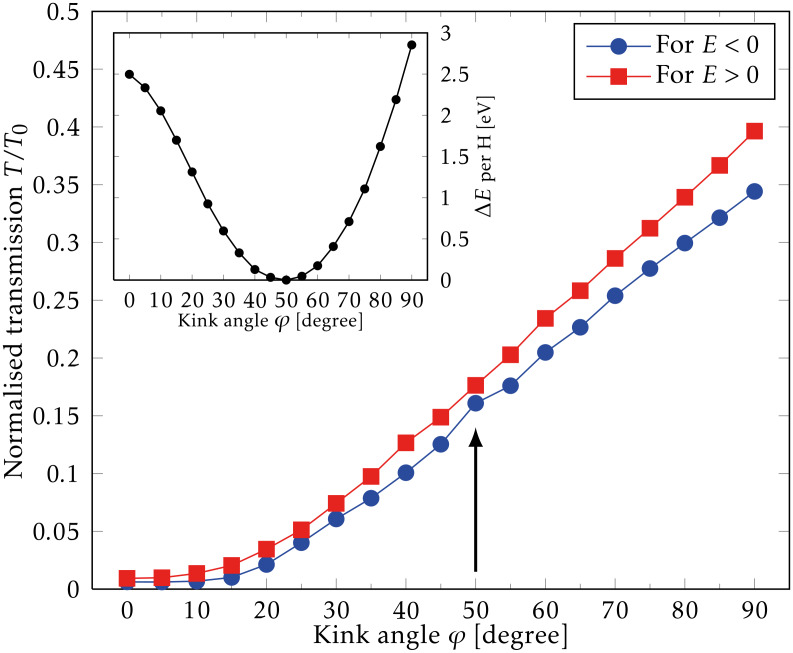
Electronic transmission through a single kink normalised by the transmission of pristine graphene (*T*_0_) as a function of the kink angle, φ, for electrons (*E* > 0) and holes (*E* < 0). The arrow indicates the normalised transmission at the equilibrium angle determined from the total energy calculations shown in the inset.

Next, we examine the energetic and transport properties of kink-lines in the armchair direction. We first consider a single kink with angle φ, e.g., between sections *S*_1_ and *S*_2_ in Figure 1b. The kink-angle φ is varied in the range 0°…90°, while the three nearest unit cells on each side of the kink are allowed to relax. The total energy per H is shown as a function of φ in the inset of Figure 3, showing a minimum energy for φ ≈ 50°. This angle roughly corresponds to the angle in an sp^3^ configuration where 2φ = 109.5°. The adsorption of H causes local changes in the geometry, i.e., only the carbon atoms very close to the kink are moved, while the remaining structure remains unperturbed. For this reason, the adsorption of hydrogen atoms can be considered as a process that locally pins the bend.

The electron transmission per unit-cell width is linear in energy for pristine graphene in a energy range around the charge neutrality point (*E* = 0), e.g., *T*_0_ 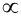 *E*. We find similarly that the calculated kink-transmission curves are also linear, and therefore we express the results for the transmissions in terms of the roughly energy-independent ratio *T*/*T*_0_ = const. The kink breaks the electron–hole symmetry, and we fit *E* > 0 and *E* < 0 separately, as shown in Figure 3. Larger kink angles result in an increase in the overall transmission, which may be attributed to a better π-orbital overlap across the kink. For the equilibrium angle, φ = 50°, the ratio *T*/*T*_0_ is close to 0.17 in both regions (indicated by the arrow in Figure 3), corresponding to a transmission reduction of 83%. Thus, we see that the hydrogen-induced kinks in graphene can be used to form effective electron barriers. We now turn to the effect of multiple barriers and periodic kink structures in order to examine resonant tunnelling phenomena and band gap formation.

### Two kinks

**Figure 4:**
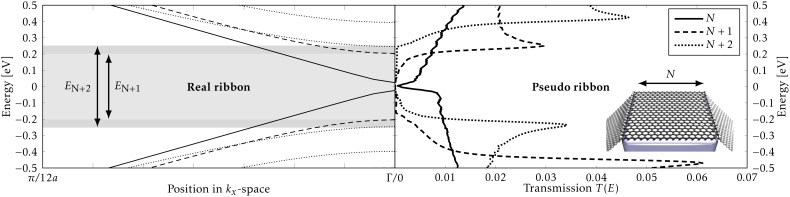
(Left) Band structures for H-passivated armchair ribbons with varying width, *N*. The ribbons are a zero-gap semiconductor, a semiconductor with band gap *E*_N+1_ = 0.4 eV, and a semiconductor with band gap *E*_N+2_ = 0.5 eV for widths *N*, *N* + 1, *N* + 2, respectively. All widths are based on the number of carbon atom lines *N* = 17. The band gaps are indicated by arrows and are highlighted. (Right) The electronic transmission functions for the corresponding pseudo-ribbons, i.e., across two parallel kinks of varied separation as shown in the inset.

Band-structure calculations show that periodic nanoscale rippling of the graphene is not sufficient to create a band gap [24] due to the low scattering by the elastic deformation [17]. In contrast, periodic arrangements of adsorbed hydrogen can indeed induce a semiconducting band gap [24,26]. The electronic band structures of hydrogen lines on flat graphene have been examined by Chernozatonskii et al. [25–26], and recently also for nanoscale-rippled graphene [24]. Here we show how two parallel kinks lead to a local electronic structure that resembles that of an isolated GNR between the kinks. Such structures could be produced experimentally by using the techniques described by Pan et al. [12]. Hydrogen-terminated armchair GNRs are semiconducting but have a small energy gap when the width in atomic lines is *N* = 3*L* − 1, where *L* is an integer [41]. In Figure 4 we compare the electronic bandstructure for armchair GNRs (aGNRs) (left panel) to the electronic transmission through two kinks separated by the corresponding aGNR width (right panel). In the present case the initial width (or, kink separation) is *N* = 17 atomic lines of carbon, which shows a semimetallic behaviour in the transmission spectrum with a small transport gap. In accordance with isolated aGNRs the next two widths *N* = 18, 19 are semiconducting, while the last investigated width *N* = 20 is semimetallic again (not shown). The close correspondence between the electronic band structure for the GNR and the transmission gap for the double-kink system allows us to consider the structure between two kinks as a pseudo-ribbon.

For the semiconducting pseudo-ribbons transport gaps surrounded by van Hove-type 1D behaviour are seen in the transmission functions (Figure 4, right panel). The transport gaps, *E*_gap_ = 0.4 eV and *E*_gap_ = 0.5 eV, are in reasonable agreement with the power-law scaling of *E*_gap_ with width found for aGNRs [41]. We note that the pseudo-ribbon breaks the electron–hole symmetry: For the *N* = 18 case a larger van Hove resonance is seen at the valence band edge, while for *N* = 19 a larger resonance is seen at the conduction band edge. There are small transmission values within the electronic band gap due to leakage through the barriers, which we expect to introduce shifts in the energies between the real and pseudo aGNR.

### Multiple kinks

**Figure 5:**
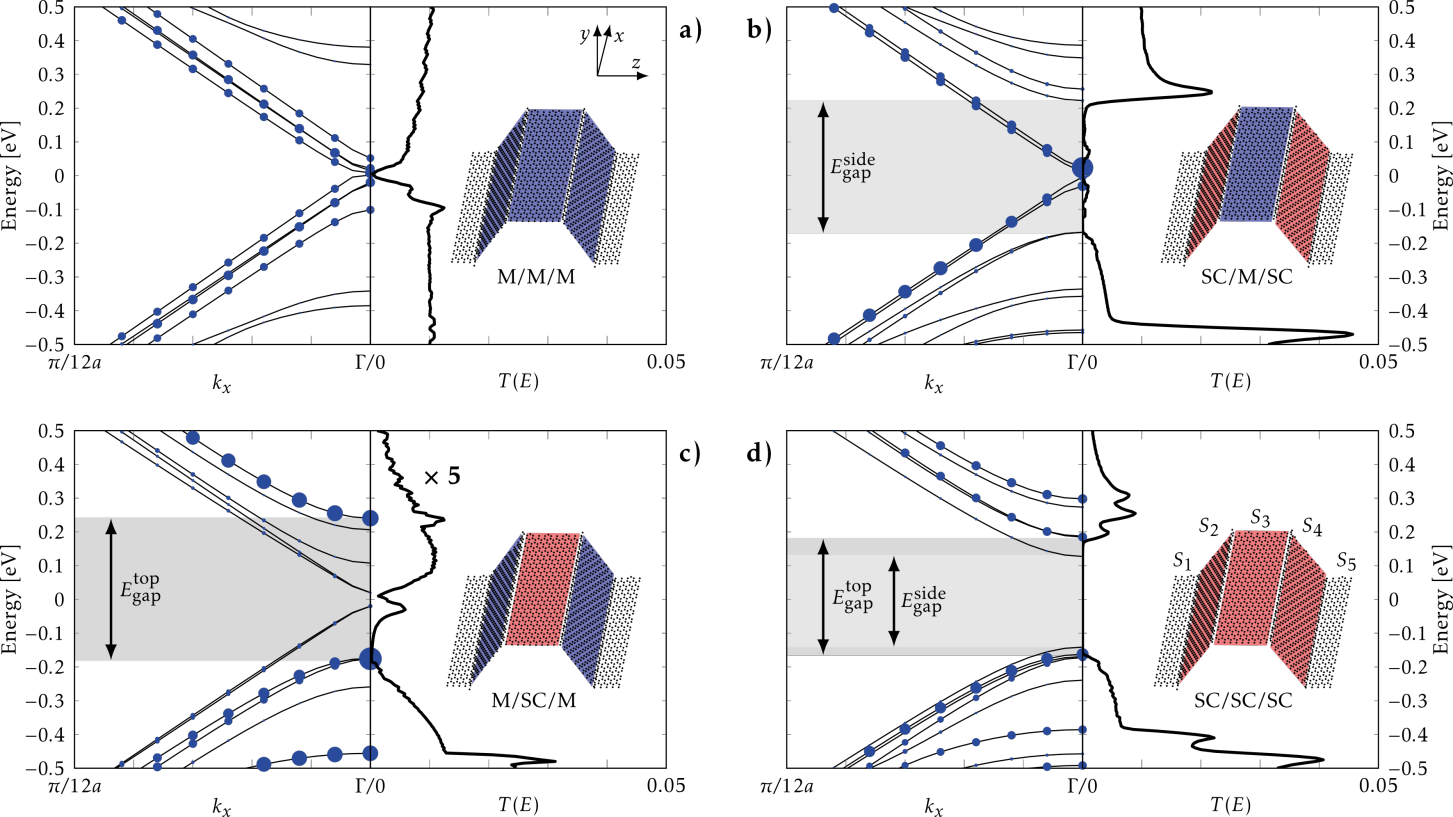
Projected band structure and transmission through structures with multiple kinks. The top (section *S*_3_) and connecting pseudo-ribbons (sections *S*_2_/*S*_4_) are varied in width, changing their electronic properties. The projected band structure along the pseudo-ribbons (i.e., *k_x_*) is shown by using filled circles with radii proportional to the weight on section *S*_3_. Semimetallic (M) pseudo-ribbons corresponding to *N* = 17 are shown in blue, while semiconducting (SC) ribbons of width *N* + 1 are shown in red. Band gaps of the top sections 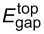 and connecting sections 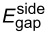 are highlighted. These are 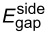 = 0.39 eV (b), 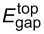 = 0.42 eV (c), and 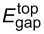 = 0.35 eV, 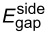 = 0.27 eV (d). The transmission *T*(*E*) (per simple transverse unitcell) is determined across all kinks in the *z*-direction, and transmission gaps comparable to the band gaps are observed in subfigures b and d.

In order to illustrate the behaviour of systems with more kinks we consider a system consisting of four hydrogen-induced kinks, as illustrated in Figure 1b and Figure 5. The sections *S*_1_/*S*_5_ are now replaced by left/right infinite-lead structures, and the “top” *S*_3_ pseudo-ribbon is connected to the leads via the “side” *S*_2_, *S*_4_ pseudo-ribbons. We keep *S*_2_, *S*_3_ identical for simplicity and determine the transmission across the kinks (in the *z*-direction in Figure 5), which is experimentally more feasible. We now investigate how the different sections influence the total transport for the four possible combinations of semiconducting (SC) and semimetallic (M), corresponding to the pseudo-ribbon widths *N*, *N* + 1 used in Figure 4.

In order to analyse the transmission we single out the band structure projected on to the top section, *S*_3_ (excluding carbon and hydrogen atoms at the kink), in the band structure along the pseudo-ribbon direction. The weight on *S*_3_ is represented by a circle of radius *R_nk_*,

[1]
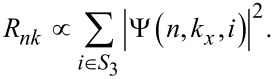


Here Ψ is the wave function at *k_x_* (*k_z_* = 0) with *n* (*i*) being the band (orbital) index. The obtained projected band structures are shown in the left parts of each subfigure in Figure 5. Generally, some bands have no weight (no circles), while others have significant weight indicating that there is little mixing between the orbitals from section *S*_3_ and other sections.

In Figure 5a and Figure 5b we consider pseudo-ribbons with semimetallic top regions, namely *S*_2_/*S*_3_/*S*_4_ being M/M/M and SC/M/SC, respectively. For the all-metal pseudo-ribbons, M/M/M, an almost energy-independent transmission function is seen with a transmission close to that of the metallic double-kink in the previous subsection. The SC/M/SC structure shows a transport gap similar to that of the single SC pseudo-ribbon with van Hove resonances, while the *S*_3_-projected band structure reveals isolated metallic states within the gap. We note that the transmission at the resonances for the SC/M/SC structure is larger than the corresponding M/M/M transmission. For the case of semiconducting top pseudo-ribbons in Figure 5c and Figure 5d, we note that M/SC/M show a greatly reduced transport gap compared to the single pseudo-ribbon case (also, note the scaling of the transmission axis), while the SC/SC/SC structure shows a complete extinction of the transmission in the electronic gap, as expected. Generally, we find that the main behaviour of the transmission is controlled by the connecting sections *S*_2_, *S*_4_, i.e., there is a good correspondence between the side section band gaps 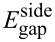 and the transmission gaps.

## Conclusion

The presented investigations show that linear kink-line structures may form in graphene by reacting with atomic hydrogen along a linear bend in the sheet. The adsorption barrier is lowered in the close vicinity of the bend, which can be exploited to form the kink. In particular, we have shown that a radius of curvature of ≈20 Å reduces the hydrogen adsorption barrier by roughly 16% compared to H adsorption on pristine graphene. The calculations suggest that once a single hydrogen atom has been adsorbed, the induced local kink and resulting increase in local curvature makes it easier for the following H to adsorb, thus creating a propagating kink formation. A full line of hydrogen atoms pins the structure and divides the electronic systems into different regions. We have shown that the electronic transmission through a single kink is reduced by 83% compared to pristine graphene, meaning that the kink-line acts as an efficient barrier for electron motion. We have demonstrated how two close-by parallel kinks form a pseudo graphene nanoribbon with similar behaviour of the electronic structure to that for isolated nanoribbons. The transmission function displays transport gap features corresponding to the isolated nanoribbon band gaps.

The present work thus suggests that it may be feasible to template functional electronic nanostructures by using the conformation of graphene, e.g., to the substrate, and that this in turn induce changes in local reactivity. Our work clearly calls for extensions in a number of directions. First of all more calculations are needed in order to investigate the kink-line propagation reaction proposed by our results. To this end it is important to include a realistic description of the actual substrate. It is also interesting to consider other adsorbate species, possibly introducing doping of the pseudo-ribbons and electronic gating. Finally, decoration and pinning of the edges of other geometries such as “bubbles” or “blisters” is of interest, e.g., in order to produce GAL-like structures [7] without perforating the graphene sheet. Calculations have shown how the adsorption of hydrogen is correlated over a length scale involving several of the unit cells employed in this work [22,35,42–43]. Thus the adatom–adatom interaction will play a significant role in the kink-line propagation along the step and will be addressed in a future study.
